# Unraveling the path to osteoarthritis management: targeting chondrocyte apoptosis for therapeutic intervention

**DOI:** 10.3389/fcell.2024.1347126

**Published:** 2024-05-17

**Authors:** Yi Ting Lee, Mohd Heikal Mohd Yunus, Muhammad Dain Yazid, Azizah Ugusman

**Affiliations:** ^1^ Department of Physiology, Faculty of Medicine, Universiti Kebangsaan Malaysia, Cheras, Malaysia; ^2^ Centre for Tissue Engineering and Regenerative Medicine, Faculty of Medicine, Universiti Kebangsaan Malaysia, Cheras, Malaysia

**Keywords:** osteoarthritis, apoptosis, autophagy, endoplasmic reticulum stress, oxidative stress, inflammation

## Abstract

Osteoarthritis (OA) is a chronic disease affecting joints and further causing disabilities. This disease affects around 240 million people worldwide. It is a multifactorial disease, and its etiology is difficult to determine. Although numerous therapeutic strategies are available, the therapies are aimed at reducing pain and improving patients’ quality of life. Hence, there is an urgent need to develop disease-modifying drugs (DMOAD) that can reverse or halt OA progression. Apoptosis is a cell removal process that is important in maintaining homeostatic mechanisms in the development and sustaining cell population. The apoptosis of chondrocytes is believed to play an important role in OA progression due to poor chondrocytes self-repair abilities to maintain the extracellular matrix (ECM). Hence, targeting chondrocyte apoptosis can be one of the potential therapeutic strategies in OA management. There are various mediators and targets available to inhibit apoptosis such as autophagy, endoplasmic reticulum (ER) stress, oxidative stress, and inflammation. As such, this review highlights the importance and potential targets that can be aimed to reduce chondrocyte apoptosis.

## 1 Introduction

Osteoarthritis (OA) is a chronic condition that affects the joints and results in abnormalities in the subchondral bone and cartilage and is one of the primary causes of disability (Peng et al., 2021; Zhong et al., 2022). The number of patients with OA-related disabilities increased by 114.5% globally between 1990 and 2019 (Long et al., 2022). According to the Centers of Disease Control and Prevention (Centers for Disease Control and Prevention, 2020), OA affects more than 32.5 million adults in the United States. Globally, around 240 million individuals have OA which limits their ability to engage in certain activities (Katz et al., 2021). Based on a review of 88 studies, females (1.69) had a higher incidence of OA than males (1.39) and the prevalence of OA rose with ageing and peaked between the ages of 70 and 79 (Cui et al., 2020). OA has been constantly on the rise in prevalence over the past 50 years as the life expectancy of people has increased (Mao et al., 2021). Moreover, it is expensive, contributing to an increase of $185.5 billion in healthcare costs between 1996 and 2005 which leads to a social and financial burden to the individual, healthcare system and society (Parmelee et al., 2015). According to data from the National Institutes of Health’s Osteoarthritis Initiative (OAI) study, those with multiple sites of OA, as well as hip or knee OA, are more likely to develop depressive symptoms (Veronese et al., 2017). Greater odds of memory loss and suicidal thoughts are found in OA patients, with these effects being primarily mediated by sleep deprivation and mood disorders brought on by joint pain (He et al., 2020). Some common symptoms of OA are pain, stiffness, popping or crepitus during joint motion, discomfort, swelling or even deformity of the joints, and mobility issues, particularly acute and fleeting morning stiffness (Fu et al., 2018).

Any joint throughout the body can develop OA, but it most commonly affects big weight-bearing joints like the knee and hip, which are characterized by reactive bone hyperplasia at the boundary of the joint and below the cartilage, thin and harsh articular cartilage, and synovial distension and inflammation (Litwic et al., 2013; Hunter and Bierma-Zeinstra, 2019). Numerous degenerative changes, including cartilage erosion and matrix breakdown, the production of pro-inflammatory mediators, chondrocyte death, and stimulation of macrophages in the synovium, developed as OA progressed (Hunter and Bierma-Zeinstra, 2019). Joint space narrowing, formation of osteophytes, subchondral sclerosis, cysts formation, and bone shape irregularities are all radiographic signs of OA (Arden and Nevitt, 2006). OA is a heterogeneous disease that affects all joint components including the ligaments, capsule, synovium, articular cartilage, and subchondral bone leading to tissue-level failure (Brandt et al., 2006; Varady and Grodzinsky, 2016; He et al., 2020). Among the degenerative changes, the degradation of cartilage is thought to be the essential characteristic of OA because articular cartilage is physically positioned in the forefront of the body’s response to the local biomechanical environment by offering a low friction system and precisely absorbing and distributing mechanical pressures imparted to the articular joint to facilitate mobility (Poole, 2012; Camarero-Espinosa et al., 2016).

There are various therapeutic strategies available for OA, such as exercise therapy, acupuncture, weight management, topical therapies, arthroscopic lavage and debridement, viscosupplementation, and pharmacological treatment using non-steroidal anti-inflammatory drugs (NSAIDs) (Lee et al., 2013; Cucchiarini et al., 2016; Luan et al., 2019; Tu et al., 2021). Yet, all the treatments are palliative and did not focus on treating or reversing the disease, and NSAIDs are chondrotoxicity and bring various side effects on organs such as the cardiovascular system, gastrointestinal system, and kidneys (Al Faqeh et al., 2012; Sukhikh et al., 2021; Shi et al., 2022). In the bargain, arthroplasties and osteotomy are still the sole approach to treating people with end-stage OA. However, this approach is intrusive and did not completely restore joint function (Xu K. et al., 2021; Mohd Yunus et al., 2022). Thus, a disease-modifying osteoarthritis drug (DMOAD) that can mitigate, halt, or reverse the development of OA is desperately needed (Hermann et al., 2018). The progression of OA does not only depend on a single cytokine alone. Instead, different cytokines can also stimulate the same signaling pathways, and the interaction between several variables is critical in the initiation and development of the disease (Molnar et al., 2021). Moreover, the direct etiology of OA is difficult to determine, but there are a few risk factors associated with OA, including ageing, genetic predisposition, obesity, trauma, inflammation, hormone profile, and excessive loading (Lee et al., 2013; Mohd Heikal et al., 2019; He et al., 2020). However, OA is known as a chronic, polygenic, and multifactorial joint condition associated with ageing (Man and Mologhianu, 2014). The possibility of developing OA greatly increase with age. During ageing, there will be change in the tissue’s mechanical and biochemical properties causing gradual buildup of damaged organelles and macromolecules in somatic cells which reduces the cells’ capacity for survival and proper function (Vellai, 2009; Peters et al., 2018). Also, the development of OA has been correlated with various proapoptotic factors that triggers apoptosis (Grogan and D’Lima, 2010). Therefore, therapies that can inhibit catabolic activities, promote anabolic activities and prevent chondrocytes death by targeting signaling pathways are the goals of OA therapies. In this study, the potential avenues for reducing chondrocyte apoptosis and the possibility of targeting apoptosis have been discussed to provide insight for targeting apoptosis in the treatment of OA.

## 2 Apoptosis

There are different types of cell death that lead to OA. These include apoptosis, necrosis and chondroptosis (Salucci et al., 2022). Chondrocyte necrosis is generally less prevalent in OA as it is acute and normally occurred when tissues are exposed to severe mechanical injuries or highly toxic substances (Del Carlo and Loeser, 2008). On the other hand, chondroptosis, a non-classical manner of chondrocyte death occurs due to unusual chondrocytes condition to eliminate cellular remnants without inflammation such as hyperthermia (Salucci et al., 2022). For this study, our focus was on investigating cell apoptosis in the context of OA treatment (Heraud et al., 2000; Musumeci et al., 2011; Thomas et al., 2011; Zamli and Sharif, 2011; Lu et al., 2012).

Apoptosis or programmed cell death is a genetically determined cell removal process that is essential in various biological processes such as normal cell renewal, immune system development and operation, embryogenesis, chemical-induced apoptosis, and hormone-depending degeneration. Apoptosis is a normal process as well as a homeostatic mechanism that occurs during development and ageing to sustain cell populations in tissue. When there is an occurrence of immune reactions, disease, or toxic chemical-induced cell damage, it will act as a defensive mechanism (Chowdhury et al., 2006; Elmore, 2007). Cell apoptosis is normally characterized by the condensation of chromatin and disintegration of nuclear in the nucleus together with pyknosis, pseudopod retraction, and rounding up of the cell (Kroemer et al., 2009). Apoptosis dysregulation results in pathological conditions such as cancer, developmental abnormalities, and degenerative illnesses (Hwang and Kim, 2015).

**FIGURE 1:**
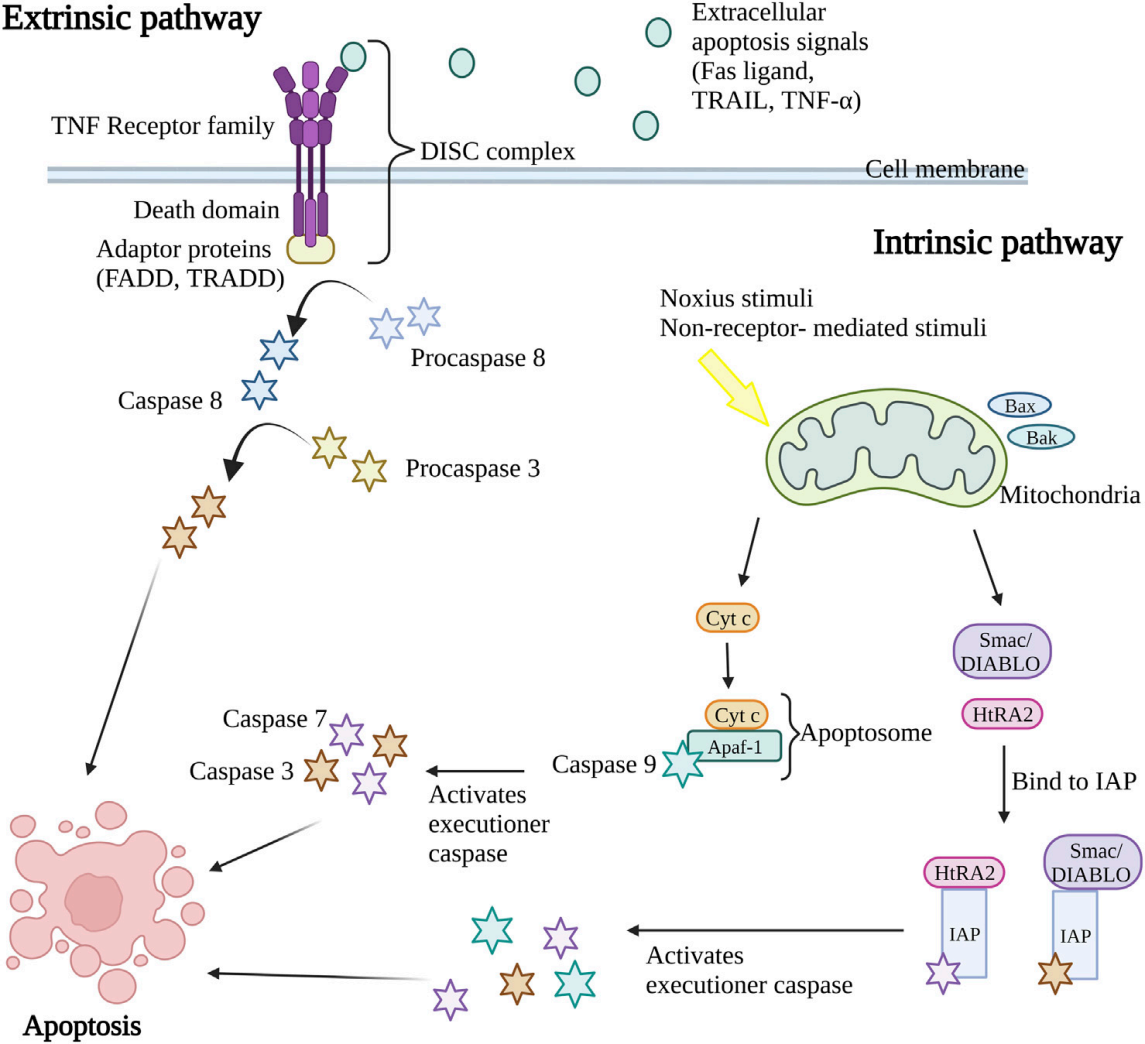
Extrinsic and intrinsic apoptosis pathway. The intrinsic pathway and extrinsic pathway are the two primary mechanisms for triggering apoptosis (programmed cell death). In the extrinsic pathway, cell surface death receptors attach to outside signals including Fas ligand, TRAIL, and TNF-α, which starts a series of events that eventually results in cell death. The intrinsic route results in the release of apoptotic molecules that activate caspases and cause cell death as a result of internal stimuli like hypoxia or toxins that cause the permeabilization of the mitochondrial outer membrane. The end outcome of both pathways is the activation of caspases, which starts nuclear apoptosis and various subsequent events that result in cell death. TNF, Tumor necrosis factor; FADD, Fas-associated death domain; TRADD, TNF receptor-associated death domain; DISC, death-inducing signal complex; TRAIL, TNF-related apoptosis-inducing ligand; Bax, Bcl-2-associated X protein; Bak, Bcl-2 antagonist killer 1; Smac/DIABLO, second mitochondria-derived activator of caspase/direct IAP binding protein; HtRA2, serine protease Omi/high-temperature requirement protein A2; cyt c, cytochrome c; Apaf-1, apoptosis protease activating factor-1; IAP, inhibitor of apoptosis protein. Created with BioRender.com.

Caspases, a group of cysteine-dependent aspartate-directed proteases are the main mediators of apoptosis through a sequence of chemical reactions (Palai and Mishra, 2015). The intrinsic (or mitochondrial pathway) and extrinsic (or death receptor pathway) pathways are the two most common apoptosis induction routes (Figure 1). Both paths finally result in a shared pathway or the apoptosis execution phase (O’Brien and Kirby, 2008). In the extrinsic apoptosis pathway, the receptors in the tumor necrosis factor (TNF) receptor superfamily such as Fas and/or TNF-related apoptosis-inducing ligand (TRAIL) receptors, TNF receptors (TNFR) and Fas (CD95) play an important role. All the receptors are characterized by a cysteine-rich extracellular domain and a cytosolic domain which together act as a death domain. The binding of extracellular apoptosis signal (Fas ligand, TRAIL, and TNF-α) to the death receptors causes the formation of a death-inducing signal complex (DISC) consisting of receptors and adaptor proteins such as Fas-associated death domain (FADD) and TNF receptor-associated death domain (TRADD) that able to recruits and activate procaspase 8. Caspase-8 further activates caspase-3, resulting in cell apoptosis (Wong, 2011; Hwang and Kim, 2015).

Nevertheless, the intrinsic apoptosis pathway is activated by intracellular signaling including non-receptor-mediated stimuli that occur during failure to maintain survival signs such as the absence of hormones, growth factors, cytokines, and presence of the noxious stimuli such as toxins, hypoxic condition, radiation, and free radicals (Subasinghe et al., 2011). The crucial event in the intrinsic apoptosis pathway is mitochondrial outer membrane permeabilization (MOMP). The stimulus will cause permeabilization of the mitochondrial outer membrane permeabilization (MOMP), causing the release of intermembrane apoptotic and apoptotic molecules such as the second mitochondria-derived activator of caspase/direct IAP binding protein (Smac/DIABLO), serine protease Omi/high-temperature requirement protein A2 (HtRA2) and cytochrome c (cyt c) in mitochondria to the cytoplasm of the cell. During the activation of the intrinsic apoptosis pathway, the Bcl-2-associated X protein (Bax) and Bcl-2 antagonist killer 1 (Bak) proteins in cytosol will translocate to mitochondria, stimulating the release of cytochrome c (cyt c). The cyt c, together with adenosine triphosphate (ATP), apoptosis protease activating factor-1 (Apaf-1), and caspase-9 form apoptosome complex that in turn activates the executioner caspases pro-caspase-3 and 7, resulting in apoptosis (Kroemer et al., 2007; Howley and Fearnhead, 2008; Hwang and Kim, 2015). At the same time, the release of second mitochondria derived activator of caspase/direct IAP binding protein (Smac/DIABLO) and serine protease Omi/high-temperature requirement protein A2 (HtRA2) disrupts the binding of inhibitor of apoptosis protein (IAP) with caspase-9, caspase-3, and caspase-7 leading to the release and activation of executioner caspases (Schimmer, 2004; Wong, 2011). Both intrinsic and extrinsic apoptosis pathway activates caspase 3 that in charge of nuclear apoptosis through the activation of activated caspase-3 splices poly (ADP-ribose) polymerase (PARP) that inactivate PARP and subsequently cause cell apoptosis (D’Amelio et al., 2010). Consequently, the caspase in the downstream pathway promotes the cleavage of deoxyribonucleic acid (DNA) repair protein, cytoskeletal proteins, protein kinases, and endonucleases family inhibitory subunits. Together, they also affect the cell cycle, signaling pathways, and cytoskeleton leading to apoptosis (Ghobrial et al., 2005).

## 3 Apoptosis and OA

In this study, chondrocytes apoptosis has been selected as the target for DMOADs as apoptosis has been thoroughly investigated and has been extensively recognized as the most significant kind of cell death. Several studies have shown that OA patients’ articular cartilage contains more apoptotic chondrocytes as compared to healthy individuals (Heraud et al., 2000; Musumeci et al., 2011; Thomas et al., 2011; Zamli and Sharif, 2011). Furthermore, one of the crucial risk factors of OA is ageing. Apoptosis, a controlled form of cell death is particularly significant in the context of ageing-related events (Lu et al., 2012). Moreover, chondrocyte death is one of the central characteristics of the cartilage of the OA patients contributing to the loss of the cartilage matrix and decreased tissue (Zamli and Sharif, 2011). Articular cartilage is known to have low turnover of extracellular matrix and cells and various tests in mature cartilage revealed only a very small fraction of proliferating cells (Caramés et al., 2010). Besides, chondrocytes are the main and only occupant cells in articular cartilage, which are crucial for controlling extracellular matrix (ECM) synthesis and turnover as well as for preserving cartilage structure (Hwang and Kim, 2015; Varela-Eirin et al., 2018). As cartilage tissue is non-innervated, avascular and lacking the characteristics of undifferentiated cells, chondrocytes only possess weak self-repair capabilities after cartilage injury (Ude et al., 2014; Abramoff and Caldera, 2020; Ahmad et al., 2020). A net loss of chondrocytes results from an imbalance between the rate of death and proliferation or differentiation, compromises the homeostasis of cartilage (Grogan and D’Lima, 2010). Hence, the loss of chondrocytes in cartilage is believed to be the leading cause of the progression of OA. This strategy could assist in the management of OA and help mitigate the long-term effects of changes brought on by ageing.

There are various apoptosis mediators, such as reactive oxygen species (ROS), nitric oxide (NO), cytokines and mechanical stress available which can induce both cartilage degradation and apoptosis. In normal conditions, chondrocytes maintain a delicate equilibrium between the synthesis and breakdown of ECM components primarily collagen type II and proteoglycan (Hosseinzadeh et al., 2016). When OA progresses, there will be an overproduction of proinflammatory cytokines such as tumor necrosis factor-alpha (TNF-α) and interleukin 1 beta (IL-1β). This condition further promotes the secretion of proteolytic enzymes by chondrocytes, including matrix metalloproteinases (MMPs) as well as a disintegrin metalloproteinase with thrombospondin motifs (ADAMTs) which brings to the breakdown of key components in ECM (Wang et al., 2011). As the matrix constituent’s breakdown overrides the synthesis of the new matrix, the homeostatic balance is disrupted, shifting the homeostasis towards catabolism. At the same time, the inflammatory mediators will recruit leukocytes to the joints and result in the production of ROS, further promoting catabolism and causing chondrocytes death (Lee et al., 2013; Hosseinzadeh et al., 2016). The apoptosis of chondrocytes and ECM degradation may remain intact and form a destructive cycle, each exacerbating the other. The ability of chondrocytes to secrete ECM components will be reduced and causing difficulties in articular cartilage repair. The continuous loss of cartilage reduces the protective ability of cartilage to joints and further stimulates OA occurrence (Musumeci et al., 2014; Fu et al., 2018). Hence, chondrocyte apoptosis may be the target for the stop or reversing the development of OA. Although the caspase-mediated proteolytic cascade is the crucial mediators of apoptosis, the onset of apoptosis is tightly modulated by numerous factors such as TNF, p53 and Bcl-2 family proteins (Chowdhury et al., 2006). Therefore, this review aims to summarize the possible route in inhibiting chondrocyte apoptosis.

Table 1 below summarizes the studies that showed the abilities of various compounds to successfully inhibit cell apoptosis via activation or inhibition of signaling pathways. This provides endless possibilities for the discovery of novel OA treatment agents and proved that the chondrocytes apoptosis pathway could be one of the targets in the development of DMOAD.

**TABLE 1:** The *in vitro*, *ex vivo* and *in vivo* evidence of the substances and their targeted pathway in inhibiting chondrocyte apoptosis.

Subject	Route of treatment	Model/cell types	Target pathway	Findings
Rapamycin (Bao et al., 2020)	Supplemented to culture media	Chondrocytes from SD rats treated with IL-18	Inhibit mTOR signaling	↑ Atg7, LC3B/LC3A ratio and Bcl-2
				↓ p62, Bax and caspase-3/9 activation
	Intra-articular injection	SD rats treated with IL-18		Exert protective effect on cartilage
				↑ autophagy
				↓ IL-18-induced chondrocyte-specific protein degradation, inflammatory factors, and apoptosis
Glabridin (Dai et al., 2021)	Supplemented to culture media	Human OA chondrocytes	Inhibit mTOR signaling	↑ COL2A1, ACAN, SOX9, PRG4, Cat and SOD, Bcl-2, and LC3-II
				↓ ROS, apoptosis rate, cleaved PARP, Bax, cleaved caspase-3, and p-mTOR
	Intra-articular injection	Male SD rats subjected to ACLT		↓ OA-induce pain, effect of apoptosis-mediated cartilage degeneration, mTOR, MMP-13 and ADAMTS5 expression
				↑ LC3-II
Quercetin (Feng et al., 2019)	Supplemented to culture media	Chondrocytes from male SD rats treated with TBHP solution	Activate SIRT1/AMPK signaling pathway	↓ apoptosis, cleavage of caspase-3, PARP, GRP78, CHOP, ATF6 expression, p-PERK/PERK and p-IRE1α/IRE1α ratio
				↑ Bcl-2, SIRT1 and p-AMPK expression
	Intraperitoneal injection	Male SD rats undergo DMM		↓ joint space narrowing, pathological changes in degeneration, erosion in articular cartilage, OARSI score, apoptotic cell death, cleaved caspase-3, 8-OHdG, CHOP, ATF6, p-PERK and p-IRE1α
				↑ SIRT1 and p-AMPK
Quercetin (Hu et al., 2019)	Supplemented to culture media	Chondrocytes from SD rats pretreated with IL-1β	Inhibit AKT/NF-κB signaling pathway, caspase-3 pathway	↓ degradation of COL2A, sGAG reduction, activation of AKT, MMP13, ADAMTS4, NO, PGE2, iNOS, COX2, p65, Bax, and caspase-3
				↑ mitochondrial membrane potential, Bcl-2, COL2A, aggrecan expression, and IκBα
	Intra-articular injection	Male SD rats subjected to medial meniscus removal and anterior meniscotibial ligament transection		↑ articular cartilage structural integrity, glycosaminoglycan synthesis, COL2A and aggrecan deposition
				↓ OARSI score, MMP13 and apoptosis
Punicalagin (Kong et al., 2020)	Supplemented to culture media	Chondrocytes from postnatal C57BL/6 mice pups treated with TBHP	Activation of autophagy	↑ HO-1, SOD1, NQO1, Bcl-2, LC3 II/I ratio, collagen type II, Atg12-5, LC3 II/I, Beclin1 and fusion of LC3 and Lamp2
				↓ Bax, cleaved caspase-3, MMP3, MMP13, ADAMTS5, and p62 expression
	Oral gavage	Male C57BL/6 mice subjected to DMM		↓ OARSI score, p62, cleaved caspase-3 expression, apoptosis, synovium thickening hypercellularity and synovitis score
				↑ LC3 II/I ratio
Artesunate (Li et al., 2019b)	Supplemented to culture media	ATDC5 differentiated into chondrocyte-like cell treated with IL-1β	Suppress NF-κB signaling pathway	↓ MMP3, MMP13, COX2, ADAMTS5, Bax, cleaved caspase-3, cleaved caspase-7, p65 protein, apoptosis cell percentage, phosphorylation IκBα and p65 and degradation of IκBα
				↑ expression of Bcl-2
	Intraperitoneal injection	Mice subjected to ACLT		↓ calcified cartilage, proteoglycan loss and OARSI score
Celastrol (Liu et al., 2020)	Supplemented to culture media	Chondrocytes form rabbit knee joint treated with Tunicamycin	Inhibit Atf6/CHOP signaling pathway (endoplasmic reticulum stress (ERs)-mediated apoptosis pathway)	↓ apoptosis rate, expression of caspase-3, caspase-6 and caspase-9, cleaved-caspase-3/pro-caspase-3, cleaved-caspase-9/pro-caspase-9, Bip, ATF6, CHOP and XBP-1
				More effective when in combination with 4-PBA, ERs inhibitor
	Intraperitoneal injection	Female Wistar rat subjected to ACLT		↓ articular cartilage injury, synovial hyperplasia, articular weak, ATF6, Bip, CHOP, XBP-1, caspase-3 and caspase-9
				More significant when in combination with 4-PBA
Theaflavin (Xu et al., 2021b)	Supplemented to culture media	Chondrocytes form C57BL/6 mice treated with TBHP	Activate Keap1/Nrf2/HO-1 pathway	↑ type II collagen, aggrecan, Nrf2 and HO-1
				↓ expression of MMP13, ADAMTS5, cleaved caspase-3, p16INK4a, nitrite, TNF-α, IL-6 and apoptosis
	Oral administration	C57BL/6 female mice subjected to DMM		↓ joint space narrowing, surface calcification, cartilage erosion, exfoliation, subchondral bone thickness, and OARSI score
Echinacoside (Lin et al., 2021)	Supplemented to culture media	Chondrocytes from mouse cartilage treated with TBHP	Inhibit PERK-eIF2α-ATF4-CHOP signaling	↓ cell apoptosis, Bax, cleaved caspase-3, p-PERK/PERK, GRP78, ATF4, p-eIF2α/eIF2α, CHOP, MMP13 and ADAMTS5 level
				↑ Bcl-2, aggrecan, collagen type II and SIRT1 level
	Intraperitoneal injection	C57BL/6 male WT mice subjected to DMM		↓ proteoglycan depletion and articular degradation, cleaved caspase-3 and OARSI score
				↑ SIRT1 and collagen type II
Morroniside (Yu et al., 2021)	Supplemented to culture media	Mouse chondrocytes subjected to IL-1β	Suppress NF-κB signaling pathway	↓ MMP13, caspase-1, NLRP3 positive cells, p-IκBα and translocation of p65
				↑ collagen type II, Ki67 expression
	Intra-articular injection	Male C57BL/6J mice subjected to DMM		↓ osteophyte formation, subchondral sclerosis, MMP13 expression and OARSI scores
Chemically modified curcumin (CMC2.24) (Zhou et al., 2020)	Supplemented to culture media	Chondrocytes from SD rats treated with SNP	Suppress NF-κB/Hif-2α pathway	↓ p65 nuclear translocation, MMP13, cleaved caspase-3, VEGF, RUNX2, p-p65, p-IκB-α, and Hif-2α
				↑ COL2A1 and Bcl-2 expression
	Intra-articular injection	Male SD rats subjected to ACLT and medial meniscus resection (MMx)		↓ cartilage degradation, synovial thickening, chondrocyte apoptosis, Mankin score, MMP13, cleaved caspase-3, VEGF, RUNX2 Hif-2α, p-p65, and p-IκB-α expression
				↑ Col2a1 synthesis
Hyperoside (Sun et al., 2021)	Supplemented to culture media	Chondrocytes from C57BL/6 mice treated with IL-1β	Suppress Nrf2/ROS/Bax/Bcl-xl axis, PI3K/AKT/NF-κB and MAPK signaling pathway, Activate Nrf2/HO-1 signaling pathway	↓ iNOS, COX2, ADAMTS5, MMP3, MMP13, ROS, Bax, cytochrome c, cleaved caspase-9, cleaved caspase-3, cell apoptosis, phosphorylation of PI3K, AKT, ERK, JNK, C-JUN, COX2, MMP3, MMP13, phosphorylation of NF-κB p65 and IκBα
				↑ collagen type II, aggrecan, SOX9 and Bcl-xl expression
	Intraperitoneal injection	C57BL/6 mice subjected to DMM		↓ proteoglycan loss, superficial cartilage destruction, cartilage erosion and OARSI score
				↑ Nrf2 positive cell
Tanshinone I (Wang et al., 2019b)	Supplemented to culture media	CHON-001 cells treated with IL-1β	Inhibit NF-κB signaling pathway	↓ apoptosis, TNFα production, MMP13, p-NF-κB, collagen type II and aggrecan degradation
				↑ Ki67-positive cells and SOX11
	Intraperitoneal injection	C57BL/6 mice subjected to ACLT		↓ cartilage degradation, cartilage erosion, fibrous cartilage, subchondral bone thickness, synovitis scores, and OARSI scores
Bardoxolone-methyl (Pang et al., 2021)	Supplemented to culture media	Chondrocytes form male SD rats treated with TBHP	Activated Keap1/Nrf2/ARE signaling pathway, activate downstream HO-1/NQO1 signaling pathway	↓ MDA, ROS, mitochondrial membrane potential depolarization cell ratio, apoptosis, MMP9, MMP13, Bax, cleaved caspase-3, and Keap1
				↑ SOD levels, aggrecan, collagen type II, Nrf2, NQO1 and HO-1
	Cartilage explant	Osteochondral tissue from SD rats treated with TBHP		↑ area of normal cartilage structure, cell number positive for collagen II and aggrecan
	Supplemented into drinking water	C57BL/6 mice subjected to DMM		↑ chondrocytes, the stands, max contact mean intensity, print area and duty cycle
				↓ cartilage destruction, massive loss of proteoglycan, and OARSI scores
Four-octyl itaconate (Pan et al., 2022)	Supplemented to culture media	C28/I2 chondrocytes treated with IL-1β	Inhibit PI3K/AKT/mTOR signaling pathway	↓ apoptotic rate, p62, cleaved caspase-3, Bax, p-AKT473, p-PI3K, p-mTOR, MMP3 and MMP13
				↑ LC3 and Beclin1
	Intraperitoneal injection	Male SD rats subjected to DMM		↑ Beclin1 and LC3II protein
				↓ OARSI scores, MMP3, MMP13, Bax, cleaved caspase-3, p62, and serum IL-6, TNF-α, MMP3 and MMP13
Neuregulin 4 (Shi et al., 2022)	Transfection using murine recombinant Nrg4 protein)	Chondrocytes from C57BL/6 mice treated with IL-1β and lipopolysaccharide	Inhibit MAPK/JNK signaling pathway	↓ apoptosis, IL-1β, IL-6, TNF-α, p-JNK and MMP13
				↑ collage type II
	Interscapular injection (Adeno-Associated virus (AAV9) encoding Nrg4)	*Nrg4* KO on C57BL/6 J background and WT C57BL/6 J male mice subjected to DMM		↓ OARSI scores, numbers of TUNEL, collagen II double-positive cells, apoptosis, IL-1β, IL-6, and TNF-α level
SIRT3 (Xu et al., 2021a)	Transfection of SIRT3 plasmid	Chondrocytes from SD rats treated with IL-1β	Inhibit PI3K/AKT/mTOR signaling	↓ expression of MMP3, MMP13, COX2, iNOS, Bax, caspase-3, caspase-9, Mff, Rab32, MTP-18 and phosphorylation level of PI3K, AKT and mTOR
				↑ collagen type II, SOX9, aggrecan, Bcl-2, Atg5, Atg7, Beclin1, LC3B and mitochondria membrane potential
	Intra-articular injection (SIRT3 overexpressing lentivirus)	Male SD rats subjected to DMM		↓ Mankin’ score, cartilage surface destruction, MMP3, cleaved caspase-3 and p-mTOR expression
				↑ proteoglycan and complete cartilage surface
MicroRNA-93 (Ding et al., 2019)	Transfection with miR-93 mimics	Chondrocytes from male C57BL/6 mice treated with LPS	Inhibit TLR4/NF-κB pathway	↑ cell viability
				↓ TNF-α, IL-1β, IL-6, TLR4, p-p65 and p-IκB-α
	Intra-articular injection (agomir-miR-93)	Male C57BL/6 mice subjected to MMT surgery		↓ TNF-α, IL-1β, IL-6, TLR4, p-p65, p-IκB-α and apoptosis

## 4 Targets to focus for chondrocytes apoptosis inhibition

### 4.1 Autophagy and apoptosis

Autophagy is a vital, multi-step lysosomal pathway degradation process of organelles and cytoplasmic proteins through tightly associated highly conserved genes known as autophagy-related genes (ATG). This process can be categorized into five phases: initiation, autophagosome nucleation, autophagosome membrane expansion and elongation, lysosome closure and fusion, and intra-vesicular product degradation (Levy et al., 2017; Xie et al., 2020). Both autophagy and apoptosis are crucial biochemical processes that maintain homeostasis in organisms and cells. Apoptosis performs its function by eliminating damaged or undesired cells, while autophagy preserves cellular homeostasis by recovering specific intracellular organelles and chemicals. The lysosomal pathway in autophagy provides benefits in cell survival as it removes unwanted organelles and, at the same time, it supplies building blocks and energy for cellular activities. Autophagy has been proven to be a protective mechanism for normal cartilage (Caramés et al., 2010). However, in specific circumstances, autophagy might result in cell death. Both apoptosis and autophagy showed complicated crosstalk with each other, and they can be triggered by the same upstream signals causing them to occur at the exact moment (Maiuri et al., 2007; Fan and Zong, 2013).

**FIGURE 2:**
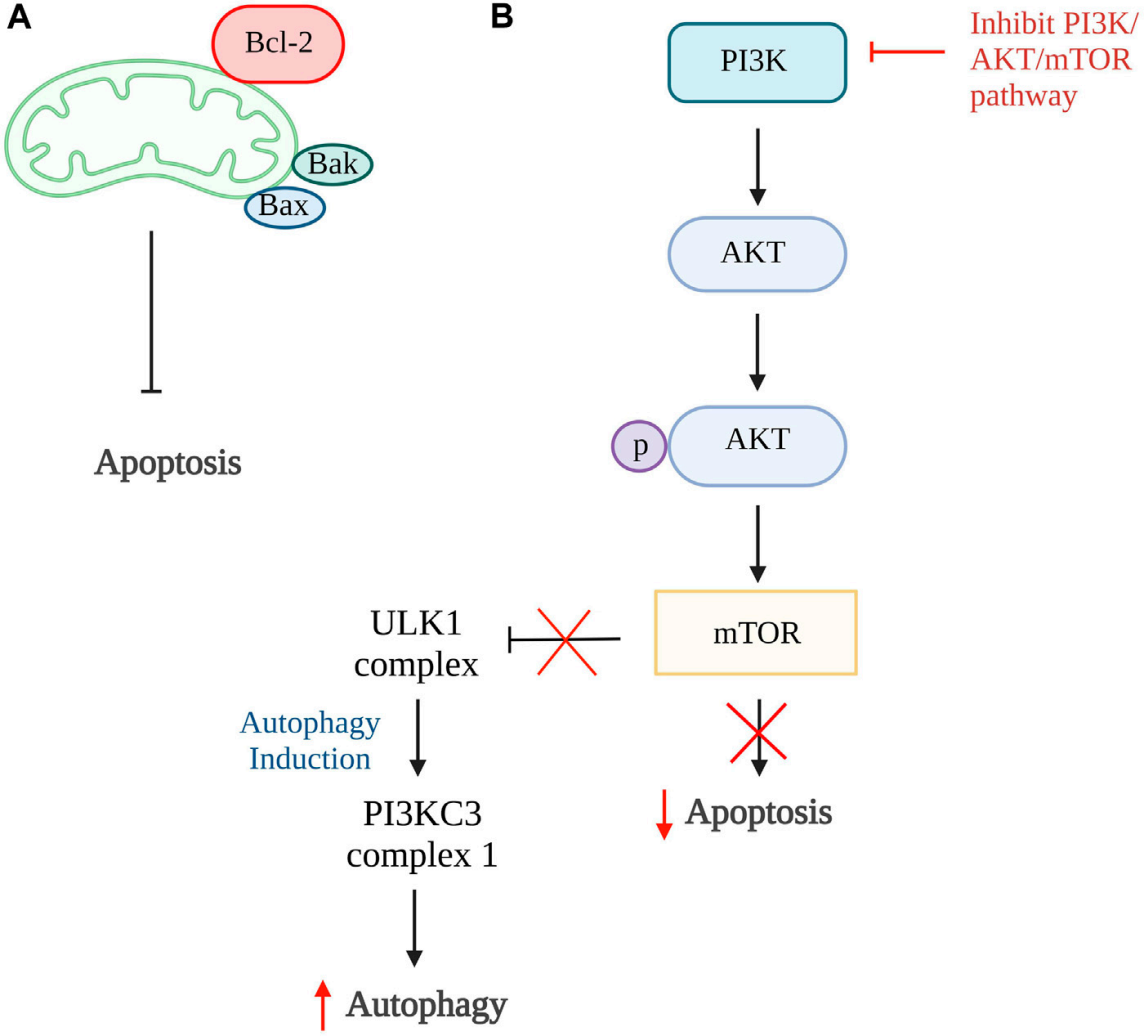
Autophagy and apoptosis. **(A)** The higher binding affinity of Bcl-2 to proapoptotic protein, Bax and Bak, inhibits the intrinsic apoptosis pathway and increases Beclin1 which promotes autophagy. **(B)** In normal condition, PI3K/AKT/mTOR pathway promotes cell apoptosis and inhibit the formation of ULK1 complex that is responsible for autophagy. Thus, the inhibition of this pathway is able to reduce cellular apoptosis and induce autophagy. Bcl-2, B cell lymphoma 2; Bax, Bcl-2-associated X protein; Bak, Bcl-2 antagonist killer 1; PI3K, phosphoinositide 3 kinases; AKT, protein kinase B; mTOR, mammalian target of rapamycin; ULK1, unc-51-like kinase 1; PI3KC3, class III PI3K complex I. Created with BioRender.com.

Autophagy and apoptosis can be inhibited by the same anti-apoptotic protein, B cell lymphoma 2 (Bcl-2). The Bcl-2 will inhibit cell apoptosis by interacting with pro-apoptotic proteins such as Bak and Bax that cause oligomerization on the outer membrane of mitochondria during apoptosis. While inhibiting cell autophagy, Bcl-2 will interact with Beclin 1, a protein only consists of Bcl-2 homology 3 (BH3), which is an important factor to trigger cell autophagy (Marquez and Xu, 2012; Fan and Zong, 2013). However, Bcl-2 has a high affinity to bind to proapoptotic protein which have multiple binding regions and thus more Beclin1 are available to increase the rate of autophagy (Figure 2A) (Shamas-Din et al., 2013). The study done by Kong et al., 2020 showed that the activation of autophagy could help inhibit chondrocyte apoptosis. Punicalagin (PUG), a polyphenol from pomegranate fruits was proven to show the ability to activate autophagy in this study. The results showed that PUG could increase the level of anti-apoptotic protein, Bcl-2, and reduce the level of apoptotic protein, Bax, and cleaved caspase-3. Moreover, the PUG showed a significant result in upregulations of essential proteins for autophagosome formation including autophagy-related protein (Atg) 12-Atg5 conjugate, microtubule-associated proteins 1A/1B light chain 3B (LC3) II/I, Beclin 1 and phosphorylation of unc-51-like kinase 1 (ULK1). The expression of autophagy receptor, p62 has also been reduced. Together, these findings revealed that PUG improves autophagy and reduces defective autophagic flux in chondrocytes. In addition, the study also proved that the activation of autophagy enhances antioxidant activity in chondrocytes and TUNEL staining further confirmed the results (Kong et al., 2020).

The phosphoinositide 3 kinases (PI3K)/protein kinase B (AKT)/mammalian target of rapamycin (mTOR) pathway can also be one of the targets in the activation of autophagy. This is because this pathway involved activation of mTOR that ameliorates autophagy activity and AKT activation, resulting in phosphorylation of caspase-9, caspase-3, and Bcl-2 associated agonist of cell death (Bad) expression that further promotes cellular apoptosis (Figure 2B) (Henshall et al., 2002; Brech et al., 2009; Xie et al., 2020). The inhibition of the PI3K/AKT/mTOR pathway and mTOR pathway alone is proven to successfully promote autophagy in a few studies (Bao et al., 2020; Xu K. et al., 2021; Dai et al., 2021; Pan et al., 2022). In the study by Pan et al., 2022, four-octyl itaconate was used and it exhibited the ability to enhance autophagy by inhibiting PI3K/AKT/mTOR pathway. Furthermore, this study showed a decrease in apoptosis rate by reducing the concentration of cleaved caspase-3 and Bax protein expression. Also, it stimulates the production of autophagy-related genes and protein expressions such as LC3 II and Beclin 1, together with the reduction of p62 receptors Similar results were found in studies done by Bao et al., 2020; Dai et al., 2021 using rapamycin and glabridin in conjunction with an increase of Atg7, Bcl-2 protein, and a reduction in cleaved PARP. Thus, these studies conclude that activation of autophagy can be the target of inhibiting apoptosis of chondrocytes.

### 4.2 Endoplasmic reticulum (ER) stress and apoptosis

The endoplasmic reticulum (ER) is a network of sacs and tubes that can be found in the cell. It is the principal site for secretory, membrane-bound, as well as some organelle-targeted protein synthesis and folding (Gorman et al., 2012). Whenever the ER is damaged by cellular stressors such as glucose deprivation, free radicals, and hypoxia, there is an increase in protein unfolding and diminished protein synthesis in the cell activating unfolded protein response (URP) (Fu et al., 2021). To counteract the negative effects of ER stress, the cells have developed a set of adaptive and defensive responses known as the UPR which is regulated by 3 ER transmembrane receptors including activating transcription factor (ATF) 6, pancreatic ER kinase (PERK) and inositol-requiring enzyme 1 (IRE1). In normal conditions, these receptors are inactivated by binding with ER chaperone, 78 kDa glucose-regulated protein (GRP78). Accumulation of unfolded proteins under ER stress causes dissociation of GRP78 that activates three ER stress receptors, initiating the UPR to eliminate unfolded protein accumulation and resume normal ER function. If the stress is not alleviated, the protective signaling will shift to a pro-apoptotic response (Schröder and Kaufman, 2005; Doyle et al., 2011; Tabas and Ron, 2011). During persistent and intense ER stress, cell apoptosis is trigger when IRE1α together with TNF receptor-associated factor 2 (TRAF2), an adapter protein, dissociates from procaspase-12 and apoptosis signal-regulating kinase 1 (ASK1) that activate c-Jun N-terminal kinase (JNK). Furthermore, IRE1α triggers the splicing of specific microRNAs, which subsequently suppresses the expression of caspase-2 and consequently promotes cell apoptosis (O’Brien and Kirby, 2008; Wong, 2011; Fu et al., 2021).

A study done by Feng et al., 2019 using quercetin, a flavonoid from fruits and vegetables able to inhibit ER stress-induced apoptosis by activating sirtuin1 (SIRT1)/adenosine monophosphate-activated protein kinase (AMPK) signaling pathway. SIRT1 is a nicotinamide adenine dinucleotide (NAD+)-dependent histone deacetylase involved in various biological processes such as DNA repair, stress responses, inflammation, and cell survival. The activation of SIRT1 can alleviate chondrocytes apoptosis by downregulating protein tyrosine phosphatase1B (PTP1B) or boosting Bcl-2 expression (Takayama et al., 2009; Gagarina et al., 2010; Haigis and Sinclair, 2010). Both the *in vitro* and *in vivo* results showed reduced in chondrocytes apoptosis number, increased Bcl-2 level and significantly reduced ER stress biomarkers, including GRP78, C/EBP-homologous protein (CHOP), ATF6 expression, phosphorylated-PERK/PERK and phosphorylated-IRE1α/IRE1α ratio. The inhibition of ER stress-induced apoptosis is proven to be related to the activation of the SIRT1/AMPK signaling pathway as the expression of SIRT1 and AMPK is increased (Feng et al., 2019).

**FIGURE 3:**
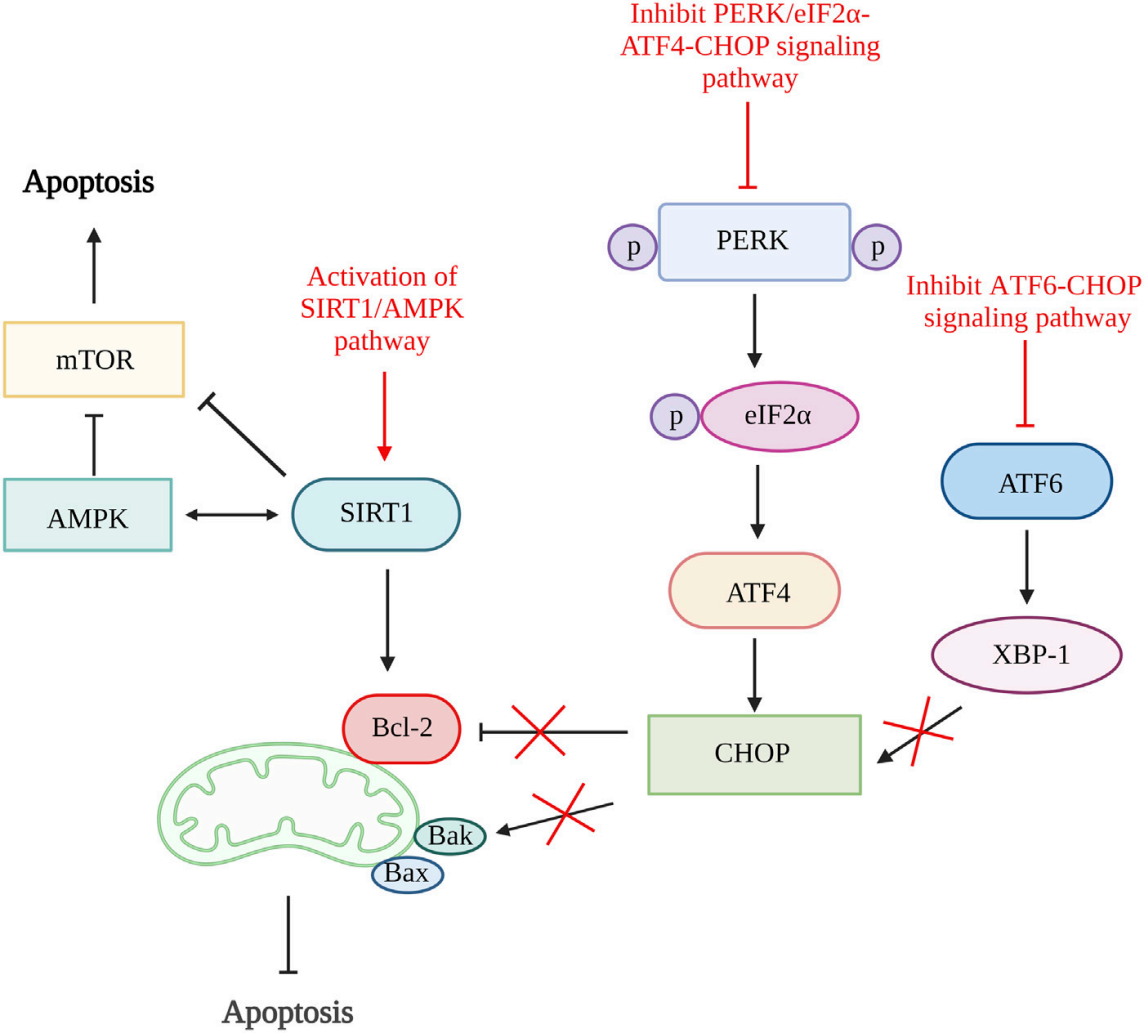
ER stress and apoptosis. The activation of SIRT1/AMPK signaling pathway increases SIRT1 that increases Bcl-2 and inhibit mTOR and further alleviate cell apoptosis. The inhibition of both ATF6/CHOP and PERK/eIF2α-ATF4-CHOP signaling pathway reduce CHOP production result in increased Bcl-2 and reduce Bak which reduce the rate of apoptosis. SIRT1, sirtuin 1; AMPK, adenosine monophosphate-activated protein kinase; mTOR, mammalian target of rapamycin; Bcl-2, B cell lymphoma 2; Bax, Bcl-2-associated X protein; Bak, Bcl-2 antagonist killer 1; PERK, pancreatic endoplasmic reticulum kinase; ATF, activating transcription factor; CHOP, C/EBP-homologous protein; XBP-1, X-box binding protein 1. Created with BioRender.com.

The inhibition CHOP related pathways such as ATF6/CHOP and PERK/eIF2α-ATF4-CHOP signaling pathway can be the target to restrain ER stress-mediated apoptosis pathway (Liu et al., 2020; Lin et al., 2021). By inhibiting the ATF6/CHOP signaling pathway, celastrol successfully lowers the apoptosis rate and reduces the expression of caspase-3, caspase-6, and caspase-9. It has been proven to reduce apoptosis rate through this pathway as it reduces the ER stress-related biomarkers such as ATF6, binding immunoglobulin protein (Bip), CHOP, and X-box binding protein 1 (XBP-1) *in vitro* and *in vivo* (Liu et al., 2020). The echinacoside also showed its ability to inhibit apoptosis by targeting the PERK/eIF2α-ATF4-CHOP signaling pathway. The results showed that echinacoside increases anti-apoptotic protein (Bcl-2, SIRT1) and reduces pro-apoptotic genes (Bac, cleaved caspase-3). Additionally, the level of p-PERK/PERK ratio, GRP78, ATF4, p-eIF2α/eIF2α ratio, and CHOP are also being reduced (Lin et al., 2021). Therefore, various studies showed that ER stress could be one of the targets in preventing chondrocyte apoptosis (Figure 3).

### 4.3 Oxidative stress and apoptosis

ROS is a product produced from a regular cellular operation. ROS are relatively unstable because of their high chemical reactivity, which promotes lipid peroxidation as well as significant protein oxidation and degradation. ROS consists of 3 major types which are hydrogen peroxide (H_2_O_2_), superoxide anion radical (O2^•–^), and hydroxyl radical (^•−^OH). The ROS can lead to cell apoptosis through 2 pathways: 1) oxidative stress that activates serine/threonine kinase AKT, extracellular signal-regulated kinases (ERK1 and ERK2), p38, and JNK and 2) DNA damage that evokes tumor suppressor gene, p53 causing transcription activation and consequently reduction in Bcl-2 expression and elevation of Bax, Apaf-1 and Fas expression (Bitomsky and Hofmann, 2009; Matés et al., 2012).

**FIGURE 4:**
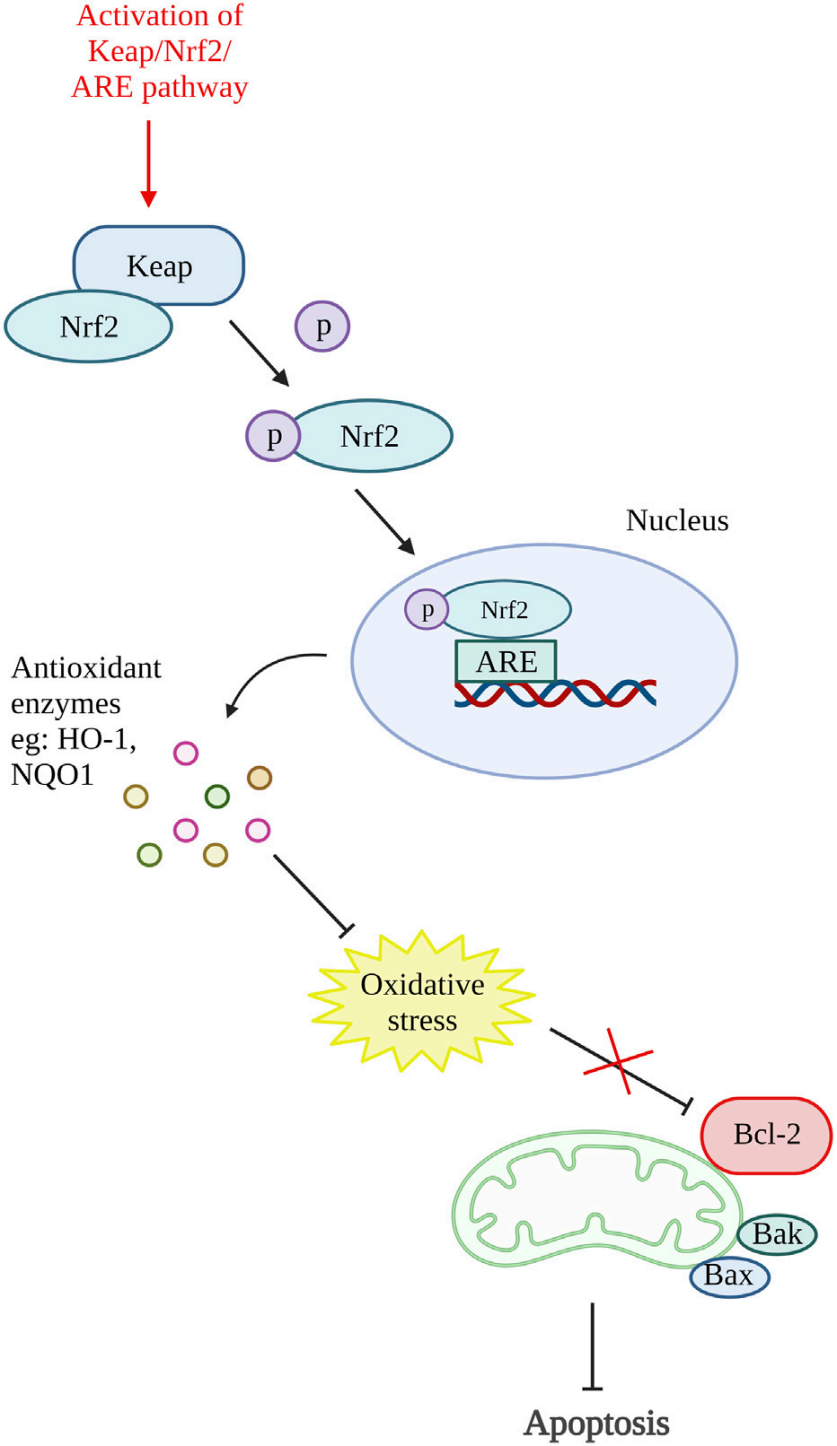
Oxidative stress and apoptosis. The activation of Keap/Nrf2/ARE pathway promotes the production of antioxidant enzymes which reduce the oxidative stress that inhibit Bcl-2. Keap, Kelch-like ECH-associated protein 1; Nrf2, nuclear factor (erythroid-derived 2)-like 2; ARE, antioxidant responsive element; HO-1, heme-oxygenase-1; NQO1, NAD(P)H-quinone oxidoreductase 1; Bcl-2, B cell lymphoma 2; Bax, Bcl-2-associated X protein; Bak, Bcl-2 antagonist killer 1. Created with BioRender.com.

A study on antioxidative response in preventing chondrocytes apoptosis was performed by Xu X.-X. et al. (2021). In this study, theaflavin (TF), a component from black tea with antioxidant properties was used to target Kelch-like ECH-associated protein 1 (Keap1)/nuclear factor (erythroid-derived 2)-like 2 (Nrf2)/heme-oxygenase-1 (HO-1) pathway. The Nrf2 is an antioxidative response mediator held in the cytoplasm by repressor Keap1. Conversely, under pathological condition, Nrf2 will be released, translocate to the nucleus and activates antioxidant responsive element (ARE)-dependent genes transcription and antioxidative enzymes production (Khan et al., 2017; Li M. et al., 2019). The *in vitro* results of this study showed that TF decreases chondrocytes apoptosis and senescence by reducing cleaved caspase-3 and senescence marker protein, p16INK4a expression. Moreover, TF is proven to inhibit apoptosis by activating the Keap1/Nrf2/HO-1 pathway as it can increase Nrf2 and HO-1 levels (Xu X.-X. et al., 2021). Another study that targeted the activation of the Keap1/Nrf2/ARE and its downstream HO-1/NAD(P)H-quinone oxidoreductase 1 (NQO1) signaling pathway using bardoxolone-methyl was carried out. A similar result with reduced ROS, Bax and cleaved caspase-3 was demonstrated by inhibiting Keap1 expression and enhancing Nrf2, NQO1 (enzymes against oxidative stress), and HO-1 expression (Pang et al., 2021). In addition, Sun et al., 2021 have also done a study targeting Nrf2/ROS/Bax/B-cell lymphoma-extra-large (Bcl-xl) axis and Nrf2/HO-1 signaling pathway by using hyperoside. The results demonstrated that hyperoside able to reduce ROS, Bax, cytochrome c, cleaved caspase-9, and cleaved caspase-3 level and thus reduce chondrocytes apoptosis. On account of that, an antioxidative response can be one of the potential therapeutic targets for preventing chondrocyte death. Figure 4 shows the illustration of the mode of action of these potential DMOADs in preventing cell apoptosis through antioxidative response.

### 4.4 Inflammation and apoptosis

Inflammation triggers many proteins and signaling pathways that are responsible for the regulation of cell death (Yang et al., 2015). In OA, various chemokines and inflammatory cytokines such as interleukin (IL)-6, IL-1β and TNFα are responsible for stimulating different inflammatory signaling pathways, including nuclear factor kappa-light-chain-enhancer of activated B cells (NF-κβ), phosphoinositide 3-kinase (PI3K)/protein kinase B (AKT) and mitogen-activated protein kinase (MAPK) pathway. The activation of the inflammatory pathway will lead to ECM degradation and the product from degradation that can enhance catabolic gene transcription while simultaneously stimulating the synthesis of inflammatory mediators via a positive feedback loop. The inflammatory pathway activation results in the release and phosphorylation of cytoplasmic p65 protein that translocate to the nucleus and amplify the gene expression of aggrecanases and degradative enzymes comprise of ADAMTS4, ADAMTS5, MMP1, MMP3, and MMP13. These result in further degradation of aggrecan and collagen. At the same time, the key pro-inflammatory and damaging mediators of OA, cyclooxygenase 2 (COX-2) and inducible nitric oxide synthase (iNOS) will also be increased, and they will upregulate nitric oxide (NO) and prostaglandin E2 (PGE2) synthesis (Kapoor et al., 2011; Rigoglou and Papavassiliou, 2013; Choi et al., 2019; Lee et al., 2022). The activation of COX has been demonstrated to increase the production of MMP3, inhibit the synthesis of collagen and proteoglycan, and accelerate chondrocyte apoptosis (Lee et al., 2013). NF-κβ is tightly regulated by various factors. Although, NF-κβ also involved in anti-apoptotic pathway, prolonged NF-κβ activation (non-canonical NF-κβ pathway) induced apoptosis through activation of various apoptosis-related mediators such as TNF, TNF-related apoptosis-inducing factor, cellular myelocytomatosis oncogene (c-myc), p53 and death receptors (Lawrence, 2009; Rigoglou and Papavassiliou, 2013; Meyerovich et al., 2016; Albensi, 2019). The activation of MAPK pathway also promotes mitochondrial-mediated apoptosis by increasing Bax/Bcl-2 ratio and result in production caspase 3 (Yuan et al., 2013).

**FIGURE 5:**
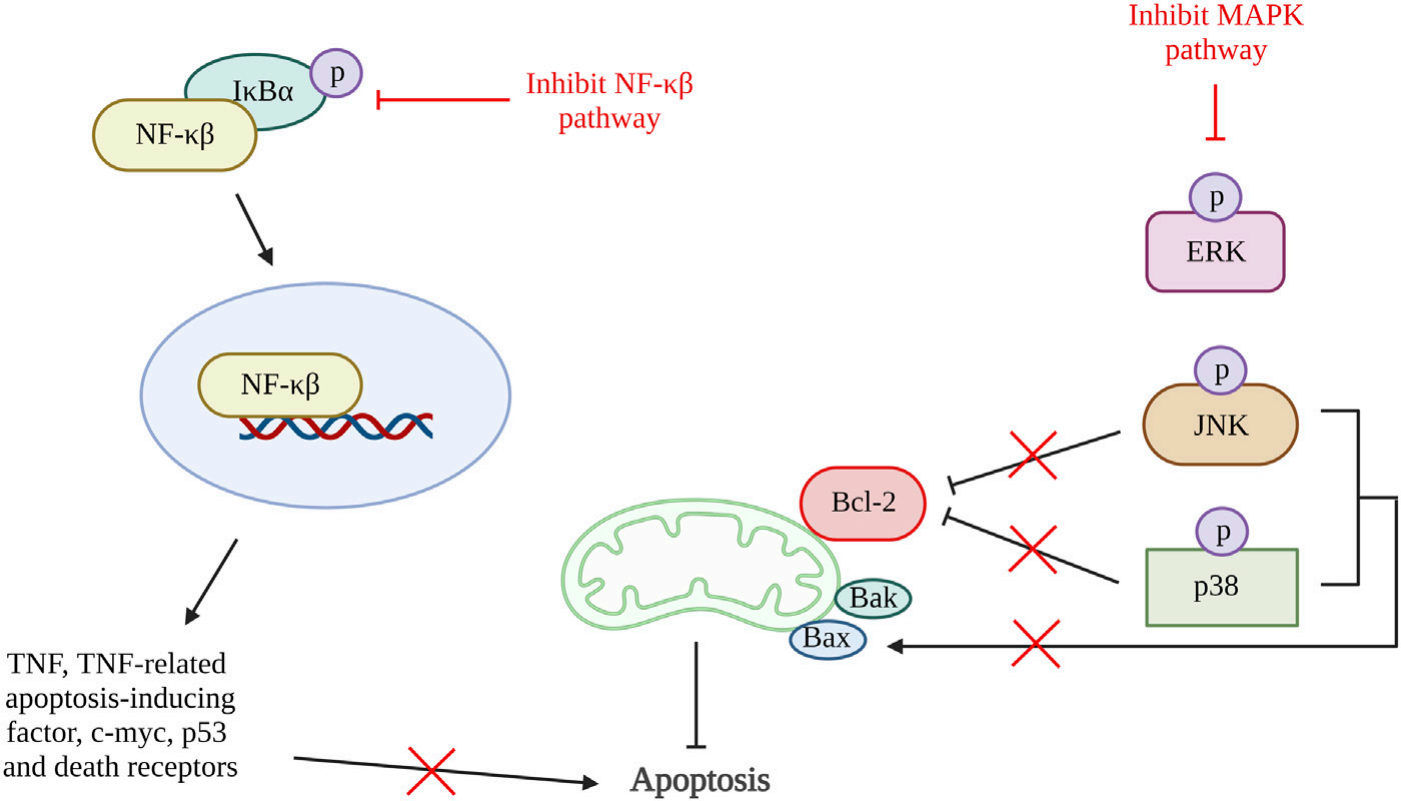
Inflammation and apoptosis. The inhibition of NF-κβ pathway reduced the production of TNF, TNF-related apoptosis-inducing factorc-myc, p53 and death receptors that reduce cell apoptosis. Activation of MAPK pathway phosphorylate JNK and p38 which inhibit Bcl-2 and activate Bax. Thus, inactivation of MAPK pathway increases Bcl-2 and reduces Bax that result in reduction of apoptosis rate. NF-κβ, nuclear factor kappa-light-chain-enhancer of activated B cells; IκBα, inhibitor of nuclear factor kappa B; MAPK, mitogen-activated protein kinase; ERK, extracellular signal-regulated kinases; JNK, c-Jun NH2-terminal kinase; Bcl-2, B cell lymphoma 2; Bax, Bcl-2-associated X protein; Bak, Bcl-2 antagonist killer 1. Created with BioRender.com.

A few studies reviewed in this article have shown that chondrocyte apoptosis can be inhibited by suppressing inflammatory pathways (Figure 5). A study using quercetin in OA treatment showed a reduction in pro-apoptotic factors, Bax, and caspase-3 and an increase in Bcl-2. It also demonstrated the reduction in PGE2 and NO production together with the decrease of AKT, an inhibitor of nuclear factor kappa B (IκBα) and p65 activation that are important in the AKT/NF-κβ signaling pathway (Hu et al., 2019). Besides that, study done by Li et al. using artesunate shows a significantly reduction in the expression of Bax, cleaved caspase-3 and caspase-7, and an increase in Bcl-2 expression through suppression of IκBα degradation, phosphorylation of IκBα and p65, and downregulate nuclear level p65 protein. A similar result was found in the study using microRNA-93, which inhibit cell apoptosis by targeting Toll-like receptor 4 (TLR4), an important NF-κβ signaling pathway regulator (Ding et al., 2019). These results are supported by a few other studies targeting the NF-κβ signaling pathway (Levy et al., 2017; Wang X. et al., 2019; Wang et al., 2019 B.; Li Y. et al., 2019; Zhou et al., 2020; Yu et al., 2021). Additionally, a study using neuregulin 4, a brown or beige adipose tissue (BAT)-enriched showed that apoptosis of chondrocytes can be halted by blocking of MAPK/JNK signaling pathway. The results showed reduction of IL-1β, IL-6, and TNF-α protein levels and mRNA expression (Shi et al., 2022). Hence, inflammation can also be one of the therapeutic targets for preventing chondrocyte apoptosis.

## 5 Discussion

A major element in the development of OA is the apoptosis of chondrocytes, which are the principal cells in charge of maintaining and repairing articular cartilage. The loss of chondrocytes impairs the ability of the remaining cells to sustain normal matrix production, which further degrades the cartilage matrix and accelerates the disease’s progression. A vicious loop that prolongs the disease is also created by the production of numerous pro-inflammatory chemicals by apoptotic cells, which can further promote cartilage breakdown and joint inflammation (Place and Kanneganti, 2019). Moreover, apoptotic cells may also cause the activation of immune cells such as macrophages, which can worsen joint inflammation and injury (Gordon and Plüddemann, 2018). As a result, creating therapeutic approaches that specifically target apoptosis is one of the key strategies for the treatment of OA.

Among various targets related to the apoptosis pathway, targeting the ER stress pathway is a promising therapeutic approach for the treatment of OA. This is because ER stress is stimulated by various stressors such as nutrient deprivation, oxidative stress, hypoxia and aging-related advanced glycation end products (AGEs) accumulation which are essential factors in causing progression of OA (Rellmann et al., 2021). Furthermore, ER stress is also an early phase in chondrocytes’ apoptosis process. Through targeting ER stress, cellular homeostasis can be restored and the effects of stressors in OA progression can be minimized. Overall, the ER stress pathway is a key regulator of apoptosis, and targeting this pathway holds great potential for treating various diseases, including OA.

Although there are various routes of administration of potential DMOADs to treat OA, intraarticular injection is recommended as the main therapeutic strategy due to several reasons including targeted distribution and better bioavailability (Jones et al., 2019). The articular cavity is less responsive to systemic medication delivery as it is avascular and innervated which limited the blood and oxygen supply (Zhang et al., 2022). Besides, the dense ECM due to organization of proteoglycans and collagen fibres of cartilage also hinders the effective delivery of drugs. Furthermore, the high concentration of negatively charged molecules consists in the ECM, makes the delivery of drug with negative charge even harder. These factors make most of the treatment modalities of OA challenging (Li et al., 2021). Also, through intraarticular injection, the therapeutics agents can be directly injected into the joint space which increases the concentration of drug to reach the pathological site as the treatment agents can bypass barriers such as liver metabolism and gastrointestinal tract. This approach helps to maximize the effectiveness of the drugs and at the same time minimize the systemic side effects (Evans et al., 2014; Emami et al., 2018).

OA is a heterogeneous disease with different phenotypes that can vary in terms of disease severity, clinical presentation, and underlying pathophysiology. Some of the common phenotypes of OA include senescent phenotype, inflammatory phenotype, metabolic phenotype, genetic phenotype and endocrine phenotype. These different phenotypes of OA may have distinct underlying mechanisms and may require different treatment approaches (Van Spil et al., 2019). However, there are various phenotypes of OA, the senescent, inflammatory, and metabolic phenotypes might be reversed by targeting chondrocyte apoptosis as the disease mechanisms involved in these phenotypes are tightly related to apoptosis of chondrocytes.

Targeting apoptosis in damaged chondrocytes is assumed to have therapeutic benefits without harming normal chondrocytes. This hypothesis is generated based on the different expression levels and activity of various apoptotic regulators between damaged and normal chondrocytes which increase the susceptibility of therapeutic compounds to damaged chondrocytes (Singh et al., 2019). Several compounds have been shown to alleviate and prevent chondrocyte apoptosis in preclinical studies, including natural compounds, antioxidants, drugs, and microRNA. The use of microRNA in OA treatment is one of the promising approaches. However, there are still many challenges such as biosafety issues, low *in vivo* efficacy, cytotoxic and high production cost (Holjencin and Jakymiw, 2022). Therefore, more efforts need to be done in future studies to make use of microRNA in treating diseases.

Currently, the field of apoptosis as a potential target is still in the preclinical stage as translating preclinical findings to a clinical setting is a complex and challenging task as there are countless factors such as safety concerns, optimization of dosage, selection of subjects, design of study and limited funding available. Hence, currently, the use of natural compounds is preferred as natural compounds are often easily accessible and cost-effective, it also has been proven to show osteoprotective and chondroprotective capabilities with fewer or no side effects (Pérez-Lozano et al., 2021). This makes them represent a promising avenue for the development of new therapeutic agents for the treatment of OA. However, more research is needed to fully understand the mechanisms of action of these natural compounds in chondrocyte apoptosis and OA progression and to determine their potential efficacy and safety in clinical settings.

## 6 Conclusion

In conclusion, this review recapitulated the possible targets for preventing chondrocyte apoptosis to alleviate the OA progression. In a nutshell, various mediators can stimulate chondrocyte apoptosis such as inflammation, oxidative stress, and ER stress. Several studies suggest that substances targeting mediators can be a potential DMOAD in halting or reversing OA progression. This study highlights the importance and potential targets that can be aimed at reducing chondrocyte apoptosis. In conclusion, this study could provide insights for researchers to further studies on OA treatment and management.
